# Preliminary Analysis of a Wireless and Wearable Electronic-Textile EASI-Based Electrocardiogram

**DOI:** 10.3389/fcvm.2021.806726

**Published:** 2021-12-20

**Authors:** Meseret N. Teferra, David A. Hobbs, Robyn A. Clark, Karen J. Reynolds

**Affiliations:** ^1^Medical Device Research Institute, College of Science and Engineering, Flinders University, Adelaide, SA, Australia; ^2^Allied Health & Human Performance, University of South Australia, Adelaide, SA, Australia; ^3^College of Nursing and Health Science, Flinders University, Adelaide, SA, Australia

**Keywords:** ambulatory cardiac monitoring, EASI ECG, electronic-textile electrodes, Holter monitoring, smart fabrics, wearable device, wearable sensors

## Abstract

**Background:** With cardiovascular disease continuing to be the leading cause of death and the primary reason for hospitalization worldwide, there is an increased burden on healthcare facilities. Electronic-textile (e-textile)-based cardiac monitoring offers a viable option to allow cardiac rehabilitation programs to be conducted outside of the hospital.

**Objectives:** This study aimed to determine whether signals produced by an e-textile ECG monitor with textile electrodes in an EASI configuration are of sufficient quality to be used for cardiac monitoring. Specific objectives were to investigate the effect of the textile electrode characteristics, placement, and condition on signal quality, and finally to compare results to a reference ECG obtained from a current clinical standard the Holter monitor.

**Methods:** ECGs during different body movements (yawning, deep-breathing, coughing, sideways, and up movement) and activities of daily living (sitting, sitting/standing from a chair, and climbing stairs) were collected from a baseline standard of normal healthy adult male using a novel e-textile ECG and a reference Holter monitor. Each movement or activity was recorded for 5 min with 2-min intervals between each recording. Three different textile area electrodes (40, 60, and 70 mm^2^) and electrode thicknesses (3, 5, and 10 mm) were considered in the experiment. The effect of electrode placement within the EASI configuration was also studied. Different signal quality parameters, including signal to noise ratio, approximate entropy, baseline power signal quality index, and QRS duration and QT intervals, were used to evaluate the accuracy and reliability of the textile-based ECG monitor.

**Results:** The overall signal quality from the 70 mm^2^ textile electrodes was higher compared to the smaller area electrodes. Results showed that the ECGs from 3 and 5 mm textile electrodes showed good quality. Regarding location, placing the “A” and “I” electrodes on the left and right anterior axillary points, respectively, showed higher signal quality compared to the standard EASI electrode placement. Wet textile electrodes showed better signal quality compared to their dry counterparts. When compared to the traditional Holter monitor, there was no significant difference in signal quality, which indicated textile monitoring was as good as current clinical standards (non-inferior).

**Conclusion:** The e-textile EASI ECG monitor could be a viable option for real-time monitoring of cardiac activities. A clinical trial in a larger sample is recommended to validate the results in a clinical population.

## Introduction

Cardiovascular Disease (CVD) is the number one non-communicable disease and the highest cause of death worldwide, with an estimated life loss of 17.92 million people in 2015 (1). This number is predicted to rise to 23.6 million by 2030 (2). Heart attack and stroke are the most common events constituting more than 85% of the CVD incidents in 2017 (1, 3).

Population aging in developed countries increases the demand for available health care. At the same time, the prevalence of CVD is also higher in this older age group, placing increased pressure on the medical system (4). A study published in 2019 reported that long-term ambulatory ECG monitoring could play a vital role in detecting the onset of ventricular dysrhythmias and atrial fibrillation (5). Ventricular dysrhythmias are the prominent factors indicating heart failure, stroke, and cardiac death. Electronic-textile-based cardiac monitoring offers a viable option (6) for long-term ambulatory monitoring outside of the hospital premises.

Electronic textiles, also known as e-textiles, are defined as “fabrics that have electronics and interconnections woven into them (7).” In the field of cardiology, researchers have developed e-textile sensors that can monitor the cardiac activities of patients while they are engaging in their day-to-day life (8) or in hospital settings (9). These e-textile ECG electrodes produce signals of acceptable quality (10) and are resistant to repeated washing in aqueous solutions without losing their properties (11, 12). However, an e-textile ECG monitor with a diagnostics capability is yet to be reported (8).

The 12-lead ECG has superior dependability and is considered the gold standard for diagnostic ECG (13). However, the conventional Holter monitor has ECG lead wires and 10 sticky electrodes, making it less comfortable for extended ambulatory monitoring. A wireless ECG monitor based on an EASI electrode configuration (14) was implemented to address this issue. This ECG monitor has a reduced number of leads (three base ECG leads; V_AI_, V_ES_, and V_AS_) and only five electrodes to realize the equivalence of a 12-lead ambulatory cardiac monitor.

## Objectives

This research focused on the testing and evaluation of ECG signals during activities of daily living with an e-textile ECG monitor with textile electrodes in an EASI configuration. The objectives of the experiments were to:

1. Examine the effect of the textile electrode characteristics (area and thickness) on signal quality
2. Investigate the effect of electrode placement on signal quality
3. Examine the effect of electrode condition due to sweating on signal quality; and
4. Compare the performance of the e-textile ECG monitor to that of a traditional Holter monitor.

## Materials and Methods

### The E-Textile ECG Monitor

**Figure 1:**
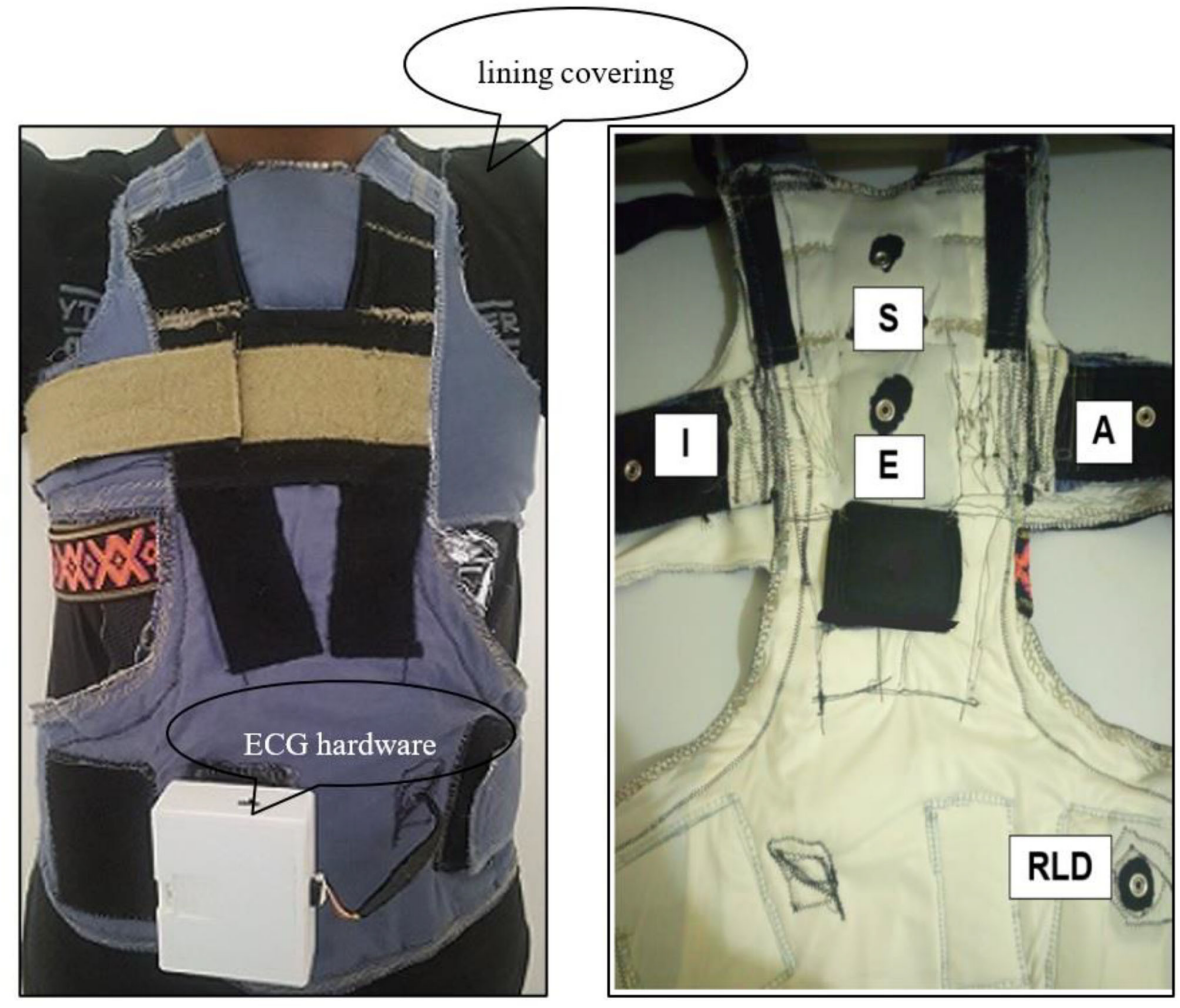
The e-textile ECG vest with textile electrodes and embedded wiring (right) the EASI electrodes attachment site on the smart ECG vest (RLD—the reference Right Leg Drive electrode) (left).

An ECG monitor consisting of a smart ECG vest, textile electrodes, and miniature ECG hardware with a Java-based real-time ECG viewer and data logger was designed. The ECG hardware weighs 152 g and has a built-in Bluetooth module that can transmit data up to a maximum distance of 100 m. The ECG monitor measures ECG using textile sensors and wiring embedded within a garment. The ECG vest has a lining covering made of a modified commercial t-shirt from K-mart (a local department store). The smart ECG vest is shown in Figure 1.

Electrodes are placed in an EASI configuration according to Feild et al. (14): (i) electrode “E” on the lower sternum at the fifth intercostal space; (ii) electrode “A” on the same level as the “E” electrode on the left mid-axillary line; (iii) electrode “I” on the same level as the “E” electrode on the right mid-axillary line; (iv), and electrode “S” at the top of the sternum, on the manubrium.

### Data Collection Protocol

The experiment was divided into two phases. During the first phase, ECGs during daily living activities (yawning, coughing, deep breathing, sitting/standing from a chair, lying on a bed in a supine position, making a call using a mobile phone, and climbing stairs) were collected from an e-textile ECG monitor to address Research Objectives 1–3. In the latter phase, the identical setups in the first stage were used to acquire ECG simultaneously from the proposed e-textile ECG monitor and a reference standard 3-leads Holter monitor (SEER light ambulatory ECG from General Electric) to answer Research Objective.

Each movement or activity was recorded for 5 min, with 2-min intervals between each recording. After each session, the lining covering of the ECG vest and the textile electrodes were replaced before the new test was conducted.

### Participants

Ethics approval to collect ECG based on the data collection protocol outlined in section Data Collection Protocol from healthy adult participants was obtained from the Flinders University Social and Behavioral Research Ethics Committee (SBREC: project code – 8490). Due to the outbreak of COVID-19 pandemic, it was not possible to collect data from members of the public. However, the protocol was adapted to a COVID safe version, and data were collected from a single healthy male volunteer (age 34, BMI 22.5 kg/m^2^).

### Signal Quality Index (SQI) Parameters

The following parameters were defined to evaluate the accuracy and reliability of the e-textile ECG monitor.

#### Signal to Noise Ratio (SNR)

Signal to Noise Ratio (SNR) measures the relative power between the desired signal and unwanted interference. SNR is one of the parameters used extensively in signal processing (15–17). SNR is defined in (1):

The equations should be inserted in editable format from the equation editor.


(1) $$\begin{array}{r} f\left(x\right)=10\log10\frac{\sum_{i=1}^{N}x{\left(i\right)}^{2}}{\sum_{i=1}^{N}{\left(x\left(i\right)-x_{r}\left(i\right)\right)}^{2}} \end{array}$$

*Where x(i)* – clean / filtered ECG signal and *x*_*r*_*(i)* – the raw ECG signal.

The higher the SNR value, the better the energy content in the e-textile ECG. For example, a lower SNR requires complex signal processing algorithms to reduce and remove noise (18).

#### Approximate Entropy

Approximate entropy (ApEn) is a statistical method used to determine the dynamic nature (randomness) of a noisy time-series signal (19). Given the variable nature of the ECG signal, the ApEn (2) of the collected data was used to study irregularities in the acquired ECG (20)^1^:


(2) $$\begin{array}{r} ApEn\left(S_{N},m,r\right)\;=\;\ln\frac{C_{m}\left(r\right)}{C_{m_{+1}\left(r\right)}} \end{array}$$

*Where:* C_m_(r) – the prevalence of repetitive patterns of length m in S_N_; S_N_ – sequence of length N; m – pattern length and r – similarity criteria.

The ApEn is interpreted differently in different disciplines. For example, lower ApEn in heart rate variability (HRV) analysis might indicate underlying pathology. However, the ApEn in the context of this study refers to the complexity and randomness of the acquired ECG, where a higher ApEn value signifies increased noise (21). The ApEn analysis was conducted for a minimum of 1,000 data points based on a similarity criterion of 0.2 and a pattern length of 2, as recommended by Pincus and Goldberger (21).

#### The Baseline Power Signal Quality Index

The baseline power signal quality index (basSQI) (3) is used to examine ECG noise artifact in the low-frequency region (f ≤ 1 Hz) as a result of deep breathing, coughing, yawning, and various body movements (16). The higher the value of basSQI, the better the signal quality. Clifford et al. (22) showed that a good quality signal had a basSQI value of 0.996 while a poor-quality ECG signal scored a basSQI value of 0.5. Therefore, a basSQI ≥ 0.95 was considered the minimum acceptable baseline low-frequency noise in this study.


(3) $$\begin{array}{r} basSQI\;=\;1-\frac{\int 0^{1}P\left(f\right)df}{\int_{0}^{40}P\left(f\right)df} \end{array}$$

#### QRS Duration and QTc Intervals

The clinical importance of the QRS duration (23, 24) and QT intervals (25, 26) to diagnose and predict possible cardiac abnormalities are well-established concepts. For an ECG monitor to have a diagnostic application, it should be able to acquire a signal with QRS duration and QT intervals equivalent to the standard 12-lead ECG (27). The QT_C_ was calculated based on the following formula (4) (27):


(4) $$\begin{array}{r} \text{QT}\text{c}\;=\;\frac{QT}{\sqrt{RR}} \end{array}$$

*Where:* RR is the time between two consecutive R peaks on the ECG tracing.

In the study, the QRS duration (normal QRS, NQRS: 0.08 – 0.12 s; long QRS, LQRS: > 0.12 s) and the corrected QT interval (QT_C_; short QT: <0.36 s; long QT: > 0.45 s) were used to evaluate the performance of the textile ECG monitor against the reference Holter monitor. If the values between the two systems were different, this was assumed to be a result of noise, so signals were then filtered using a MATLAB-based 2nd order high pass Butterworth filter (fc = 0.67 Hz) to remove the noise, and the results were again compared.

Three peak detection algorithms [Pan and Tompkins, State-Machine, and Multilevel Teager Energy Operator (MTEO)] from BioSigKit (28), a MATLAB toolkit for Bio-Signal analysis, were used to detect the Q, R, S, and T waves of the acquired ECG.

### Electrode Characteristics

**Figure 2:**
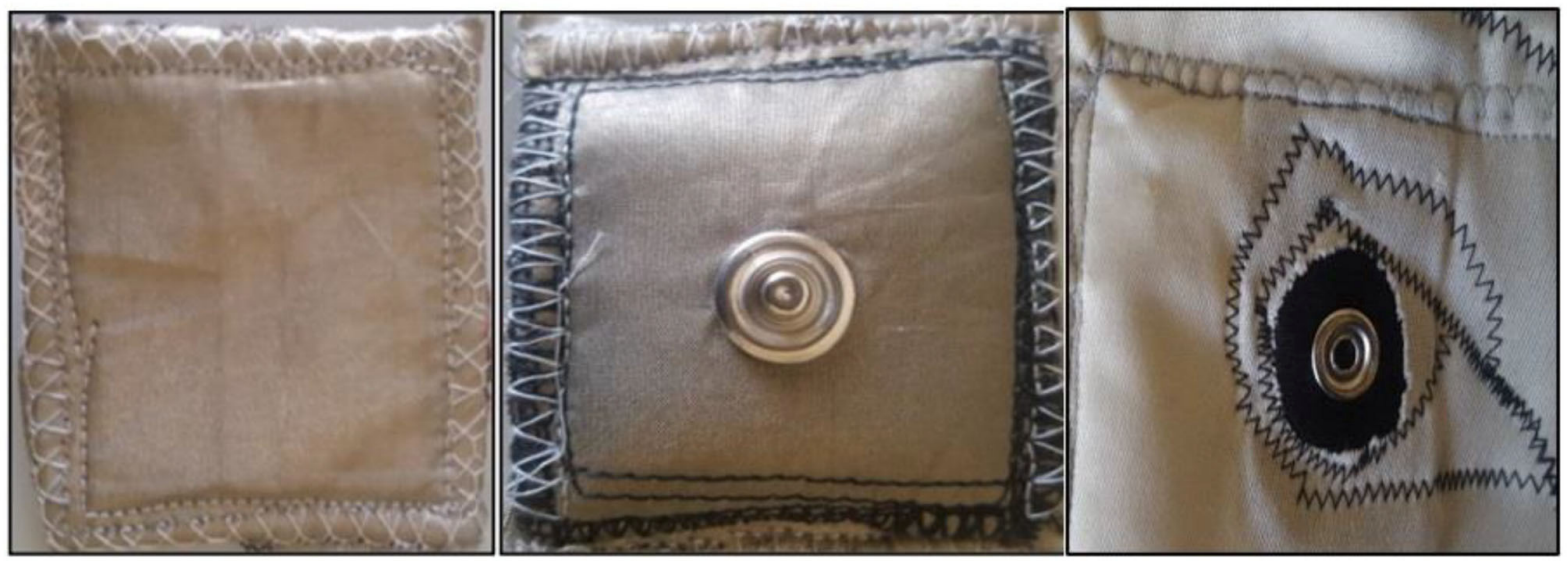
The e-textile ECG electrodes; left—front view (user side), middle—back view with male snap fastner (attached to the ECG vest) and right—textile electrode attachment female snap fastner on the ECG vest.

The relation between the noise introduced during a series of controlled movements and activities of daily living and the textile electrodes surface area was studied. Custom textile electrodes (Figure 2) of different contact surface areas (40, 60, and 70 mm^2^) were produced in the Medical Device Research Institute laboratory at Flinders University, South Australia. These were constructed from squares of silver-plated nylon conductive fabric (Adafruit Industries, New York, U.S.) sewn onto 3 mm thick Statfree® conductive polyurethane foam. Then, ECGs were acquired from these textile electrodes to investigate the effect of electrode surface area on signal quality. The thickness of the electrodes was kept at 3 mm throughout this study.

In a second experiment, the effect of electrode thickness (padding of the textile electrodes) on ECG signal transduction was assessed. 60 mm^2^ squares of silver-plated nylon conductive fabric were sewn onto 3, 5, and 10 mm thick Statfree® conductive polyurethane foam to produce padded textile-electrodes of 3, 5, and 10 mm thickness. ECGs were collected with the standard EASI electrode configuration throughout the experiment.

### Electrode Placement

Optimal electrode placement remains an active area of research in cardiac monitoring (8, 29). As the textile electrodes are not attached to the skin firmly, it is possible for the textile electrodes to move during certain activities. It was, therefore, necessary to study the effect of electrode position on signal quality. The lateral electrodes (A and I) were more likely to be knocked or moved during activity. Hence, we wanted to see the effect of varying the electrode position. Therefore, in the experiment, the positions of the two textile sensors at “A” and “I” were varied into three positions—anterior axillary, mid-axillary, and posterior-axillary and at the level of the “E”-electrode while keeping the “E” and “S” electrodes at their defined positions. Throughout the experiment, 70 mm^2^ textile electrodes of 3 mm thickness were used.

### Electrode Condition

An experiment was conducted to investigate the effect of moisture from sweating on signal quality. Signals from “wet” and “dry” electrodes were also compared to those from standard commercial Ag/AgCl electrodes.

An initial ECG was obtained with the participant in a relaxed seated position. The electrodes were considered to be dry for this measurement since the effect of sweating was minimal over this time. Once ECG acquisition from the dry textile electrodes was complete, the volunteer performed casual walking for 5 min wearing the smart ECG vest to induce sweating. ECG was then collected from the “wet” textile electrodes during different body movements and activities of daily living.

Before the start of each movement/activity, the subject rested for 5 min and was given a fresh hand towel to use to dry body sweating. Then, the textile electrodes used in the previous test were replaced with new dry textile electrodes. For the entire test duration, the standard EASI configuration and 3 mm thick, 70 mm^2^ textile electrodes were used to collect ECG.

Finally, ECGs from wet-gel electrodes (Nissha Medical Technologies, NY, United States) were collected using the e-textile ECG monitor during different body movements (yawning, deep breathing, sideways, and up movements) and activities of daily living (sitting/standing from a chair and climbing stairs) and signal quality was compared to that of the 3 mm thick 60 mm^2^ textile electrodes.

### Comparing the E-Textile ECG to the Traditional Holter Monitor

ECG was acquired simultaneously from the e-textile ECG monitor (using 3 mm thick, 70 mm^2^ textile electrodes) and from a reference standard 3-lead Holter monitor (SEER light ambulatory ECG from General Electric Healthcare, Chicago, Illinois, U.S.; using wet-gel commercial electrodes). According to the reference Holter monitor user documentation, channel one is the reference lead and is used to acquire modified V5 (mV5). A modified V1 (mV1) is obtained through channel two. It is possible to collect either modified V3 (mV3), modified aVF (maVF), or modified Z (mZ) ECG based on the lead placement connected to channel three (30)^2^. The modified maVF arrangement was selected as the lead placement during the maVF ECG does not coincide with any of the EASI ECG electrode positions.

### Data Analysis

ECG from the e-textile monitor was sampled at 4,000 samples per second (sps), recorded at 200 sps with a frequency range from 0 to 100 Hz at −3 dB level, and wirelessly transmitted to a host PC. Data were analyzed using MATLAB^TM^ 2017Ra software (The MathWorks Inc., Natick, MA, U.S.).

The ECG collected from the SEER Holter monitor was exported to MIT Signal Format upon completing the experiments. The data were then converted to an excel file (CSV UTF comma delimited—^*^.csv) and Text (Tab delimited—^*^.txt) format using a MATLAB script for ease of manipulation. According to the header file, the ECG from the Holter monitor was recorded at 125 Hz. However, the proposed textile ECG monitor has a recording frequency of 200 samples per second. Therefore, the Holter EC was resampled to match the 200 Hz rate of the textile ECG.

To retain as much low-frequency noise as possible while removing the DC offset from the inadequate skin-electrode interface and the electrode half-cell potential (31), a first-order Butterworth high pass filter (Fc = 0.067 Hz) was used to block the zero-frequency interference into the acquired ECG signal.

### Statistical Analysis

Wilcoxon Signed-Rank Test is the non-parametric form of the paired-sample *t*-test used to analyze samples where the data has unknown distribution. The Wilcoxon Test ranks the absolute values of the differences between the paired data in the two samples. It computes statistical values based on the number of negative and positive differences. If the resulting *p*-value is small (*p* < 0.05), it is safe to assume that the two samples have different distributions and reject the null hypothesis (32). In cardiac research, previous studies (13) validated the Wilcoxon Signed-Rank Test as a practical statistical tool to analyze ECG. As a result, the Wilcoxon Signed-Rank Test was used to compare results throughout the experiment. A *p*-value of < 0.05 was considered statistically significant.

## Results

Results from the e-textile ECG experiments are presented in four themes below (A) Textile electrode characteristic, (B) Electrode placement, (C) Electrode condition, and (D) Comparison between the e-textile ECG monitor and the commercial Holter monitor.

### Textile Electrode Characteristic

#### Effect of Electrode Area on Signal Quality

**Figure 3:**
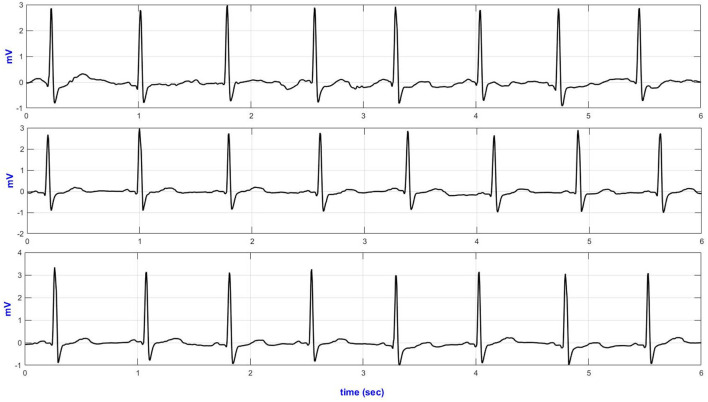
Lead-II representative ECG traces from different size textile electrodes acquired during climbing stairs (top–40 mm^2^; middle–60 mm^2^ and bottom–70 mm^2^ textile electrodes).

Figure 3 illustrates representative ECG strips from different size textile electrodes. Table 1 presents the SQI (ApEn, basSQI, and SNR) analysis results of experiments conducted to investigate the effect of electrode surface area on motion artifact.

**Table 1:** Results of SQI analysis based on lead-II ECGs from different size textile electrodes (40, 60, and 70 mm^2^).

**Body movement / activities**	**ApEn descriptive statistics (Mean** **±** **SD)**			**basSQI**			**SNR**		
	**40 mm^**2**^**	**60 mm^**2**^**	**70 mm^**2**^**	**40 mm^**2**^**	**60 mm^**2**^**	**70 mm^**2**^**	**40 mm^**2**^**	**60 mm^**2**^**	**70 mm^**2**^**
Yawning	**0.063** ± 0.0081^*^	0.049 ± 0.0049^*^	0.043 ± 0.0030	0.9661	***0.9312***	0.9946	5.2402	5.4238	6.2582
Deep Breathing	**0.048** **±** 0.0078^*^	0.046 ± 0.0067^*^	0.041 ± 0.0040	0.9791	0.9780	0.9840	5.7759	8.0175	7.7144
Coughing	**0.048** **±** **0.0081**	0.036 ± 0.0067^*^	0.044 ± 0.0039	0.9950	0.9920	0.9692	6.3124	7.3790	7.6144
Sideways	**0.061** **±** 0.0063^*^	0.055 ± 0.0080	0.055 ± 0.0055	0.9852	0.9880	0.9876	6.4403	7.8228	11.7176
Up	**0.198** **±** 0.0251^*^	0.121 ± 0.0182^*^	0.098 ± 0.0155	***0.7760***	0.9895	0.9937	5.6869	6.9605	7.2274
Sitting/Standing	**0.049** **±** 0.0057^*^	0.046 ± 0.0063	0.043 ± 0.0029	0.9812	0.9921	0.9943	7.8956	7.9628	9.1100
Stairs	**0.060** **±** 0.0091^*^	0.046 ± 0.0063	0.045 ± 0.0029	0.9828	0.9968	0.9921	7.8110	7.4803	8.7859

*Sideways, moving the hands sideways and moving them back to the midline horizontally; up, raise arms above the head and moving them back; sitting / standing, sitting / standing from a chair; stairs, climbing stairs; ApEn, Approximate entropy; basSQI, baseline power signal quality index; SNR, Signal to Noise Ratio; Bold italic - Red, moderate to intense lower frequency noise (basSQI <0.95); Bold, higher ApEn value*.

* *Statistically significant (p < 0.05) compared to the ECG from the 70 mm^2^ textile electrodes*.

The ECG from the 70 mm^2^ textile electrodes showed lower ApEn during yawning, deep breathing, and up movement and climbing stairs. In contrast, the ECG from the 60 mm^2^ textile electrodes showed lower ApEn during coughing. The ECG acquired from the 70 and 60 mm^2^ textile electrodes did not show significant ApEn difference during sideways movement, sitting/standing from a chair and climbing stairs.

Table 1 also compares the SNR values of the ECGs obtained from textile electrodes of three different surface areas (40, 60, 70 mm^2^). During deep breathing, the lead-II ECG from the 60 mm^2^ showed increased SNR. Acquiring ECG through the 70 mm^2^ textile sensors showed higher SNR compared to the smaller area textile electrodes.

#### Effect of Electrode Thickness (Electrode Padding) on Signal Quality

The signal quality parameters were computed to examine the influence of electrode thickness on the ECG signal quality during different body movements and activities of daily living. The results are presented in Table 2. As shown in Table 2, the ECG collected from the 10 mm thick textile electrodes performed poorly for all movements and activities (higher ApEn, lower basSQI, and lower SNR).

**Table 2:** Results of SQI analysis based on lead-II ECGs acquired from 3, 5, and 10 mm thick Textile electrodes.

**Body movement / activities**	**ApEn descriptive statistics (Mean ± SD)**			**basSQI**			**SNR**		
	**Thickness of the textile electrodes**								
	**3 mm**	**5 mm**	**10 mm**	**3 mm**	**5 mm**	**10 mm**	**3 mm**	**5 mm**	**10 mm**
Yawning	0.043 ± 0.0030	0.048 ± 0.0075	0.053 ± 0.0065^*^	0.9680	0.9624	***0.9138***	8.0865	5.7944	1.8421
Deep breathing	0.040 ± 0.0039	0.050 ± 0.0055^*^	0.054 ± 0.0061^*^	0.9863	0.9655	***0.9182***	7.9564	5.7428	2.7611
Coughing	0.043 ± 0.0048	0.049 ± 0.0050^*^	0.074 ± 0.0096^*^	0.9709	0.9559	***0.8619***	7.6567	5.8650	2.9173
Sideways	0.054 ± 0.0057	0.061 ± 0.0068	0.080 ± 0.0077^*^	0.9921	0.9655	***0.8074***	11.4961	6.9296	1.9336
Up	0.096 ± 0.0202	0.146 ± 0.0205^*^	0.175 ± 0.0316^*^	0.9942	0.9712	***0.9279***	7.2397	6.2622	3.8354
Sitting/standing	0.042 ± 0.0031	0.053 ± 0.0043^*^	0.081 ± 0.0102^*^	0.9953	0.9743	***0.8727***	8.4876	7.5886	4.2407
Stairs	0.046 ± 0.0025	0.056 ± 0.0043^*^	0.124 ± 0.0158^*^	0.9926	0.9692	***0.8028***	8.7658	6.2901	3.8456

*Sideways, moving the hands sideways and moving them back to the midline horizontally; up, raise arms above the head and moving them back; sitting / standing, sitting / standing from a chair; stairs, climbing stairs; ApEn, Approximate entropy; basSQI, baseline power signal quality index; SNR, Signal to Noise Ratio; Bold italic - Red, moderate to intense lower frequency noise (basSQI <0.95); Bold, higher ApEn value*.

* *Statistically significant (p < 0.05) compared to the ECG from the 3 mm textile electrodes*.

### Electrode Placement

**Figure 4:**
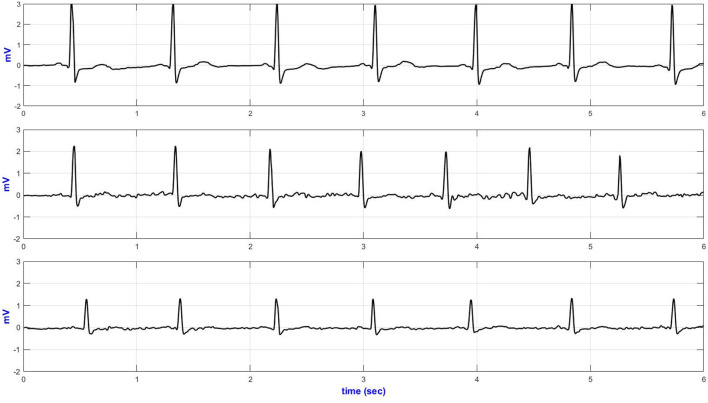
Lead-II representative sideways ECG (top—Anterior; middle—medial and bottom—posterior AI electrodes placement).

Figure 4 presents the temporal plots of lead-II ECG acquired during sideways movement.

The basSQI, average R-peak amplitude and average ECG power were calculated to assess the power characteristics of the acquired ECG and are summarized in Table 3. Placing the textile electrodes far from the heart, on the posterior-axillary lines, reduced the amplitude of the collected ECG. Therefore, even though the medial placement showed higher noise in the low-frequency range, the basSQI values were better compared to the posterior placement (Table 3).

**Table 3:** Summary of the ECG characteristics based on “AI” electrodes placement.

**Body movement / activities**	**basSQI**			**Average R-wave amplitude (mV)**			**Average ECG power (mW)**			**ApEn descriptive statistics (Mean** **±** **SD)**		
	**Electrode placement**											
	**Ant**	**Med**	**Post**	Ant^**^	**Med**	**Post**	**Ant**	**Med**	**Post**	**Ant**	**Med**	**Post**
Yawning	0.990	0.986	***0.872***	3.08	1.89	1.13	269.1	105.3	41.3	0.039 ± 0.009	0.044 ± 0.011	**0.060** **±** 0.008^*^
Deep breathing	0.988	0.987	***0.923***	3.15	2.01	1.23	258.1	105.4	40.6	0.039 ± 0.005	0.044 ± 0.007	**0.056** **±** 0.009^*^
Coughing	0.995	0.998	0.992	3.27	2.12	1.35	304.6	140.5	59.1	0.032 ± 0.005	0.038 ± 0.005^*^	**0.062** **±** 0.006^*^
Sideways	0.992	0.990	0.965	3.04	2.07	1.26	221.1	110.2	43.2	0.039 ± 0.004	**0.159** **±** 0.035^*^	0.139 ± 0.031
Up	0.993	0.992	0.964	2.89	1.93	1.24	228.8	119.6	49.4	0.074 ± 0.001	**0.384** **±** 0.059^*^	0.168 ± 0.027^*^
Sitting/standing	0.998	0.984	0.972	3.16	2.26	1.38	275.3	138.4	55.5	0.039 ± 0.005	**0.067** **±** 0.011^*^	0.065 ± 0.009
Stairs	0.998	0.991	0.981	3.08	1.89	1.13	269.1	105.3	41.3	0.038 ± 0.004	**0.087** **±** 0.013^*^	0.074 ± 0.006^*^

*Sideways, moving the hands sideways and moving them back to the midline horizontally; up, raise arms above the head and moving them back; sitting / standing, sitting / standing from a chair; stairs, climbing stairs; basSQI, baseline power signal quality index; ApEn, approximate entropy; Ant, AI electrodes placed at the left and right anterior axillary lines, respectively; Med, AI electrodes placed at the left and right medial axillary lines, respectively; Post, AI electrodes placed at the left and right posterior axillary lines, respectively; Bold italic - Red, moderate to intense lower frequency noise (basSQI <0.95); Bold, higher ApEn value*.

** *Higher R-wave amplitude*.

* *Statistically significant (p < 0.05) compared to the ECG from the Anterior AI textile electrodes placement*.

ApEn analysis was conducted to confirm that the higher power content of the ECG acquired from the anterior axillary lines is, in fact, mainly from the ECG signal, and the results are summarized in Table 3. The anterior axillary electrode placement resulted in lower randomness in the acquired signal for the entire experiment. On the other hand, the ECG obtained from the medial-axillary lines showed a higher noise level for every test involving hand movement (sideways, up, sitting/standing from a chair, and climbing stairs).

### Electrode Condition

#### Effect of Sweating on Signal Quality

The ApEn analysis results presented in Table 4 revealed that the ECG collected from dry textile electrodes exhibited higher randomness (higher ApEn) compared to the ECGs from wet textile sensors. The basSQI and SNR computation results of the ECG from dry and wet textile electrodes (Table 4) showed that the ECG acquired from the wet textile electrodes showed reduced noise in the low-frequency region.

**Table 4:** SQI analysis results of lead-II ECG from textile electrodes and commercial wet-gel electrodes using textile ECG monitor.

**Body movement / activities**	**ApEn descriptive statistics (Mean** **±** **SD)**			**basSQI**			**SNR**		
	**Dry textile electrodes**	**Wet textile electrodes**	**Wet-gel electrodes**	**Dry textile electrodes**	**Wet textile electrodes**	**Wet-gel electrodes**	**Dry textile electrodes**	**Wet textile electrodes**	**Wet-gel electrodes**
Yawning	**0.059** **±** 0.004^*^	0.038 ± 0.006	**0.058** **±** 0.005^*^	***0.9051***	0.9822	0.9614	4.6507	7.6812	6.6747
Deep breathing	**0.051** **±** 0.006^*^	0.047 ± 0.004	0.039 ± 0.006	***0.9060***	0.9900	0.9860	3.8205	7.1519	7.6097
Sideways	**0.057** **±** 0.007^*^	0.045 ± 0.005	**0.061** **±** **0.010**	0.9858	0.9932	0.9701	6.7089	11.8897	7.7883
Up	**0.181** **±** 0.019^*^	0.094 ± 0.012	**0.186** **±** 0.019^*^	0.9633	0.9944	***0.9447***	6.4031	8.3891	7.6526
Sitting/standing	**0.067** **±** 0.006^*^	0.043 ± 0.003	**0.063** **±** 0.010^*^	***0.9044***	0.9953	0.9581	6.9461	9.1111	9.3364
Stairs	**0.075** **±** 0.005^*^	0.045 ± 0.003	0.059 ± 0.007^*^	***0.9313***	0.9926	0.9733	6.9189	8.9621	9.6773

*Sideways, moving the hands sideways and moving them back to the midline horizontally; up, raise arms above the head and moving them back; sitting / standing, sitting / standing from a chair; stairs, climbing stairs; ApEn, Approximate entropy; basSQI, baseline power signal quality index; SNR, Signal to Noise Ratio; Bold italic - Red, moderate to intense lower frequency noise (basSQI <0.95); Bold, higher ApEn value*;

* *Statistically significant (p < 0.05) compared to the ECG from the wet textile electrodes*.

#### Comparison Between the Textile Electrodes and the Disposable Wet-Gel Electrodes

**Figure 5:**
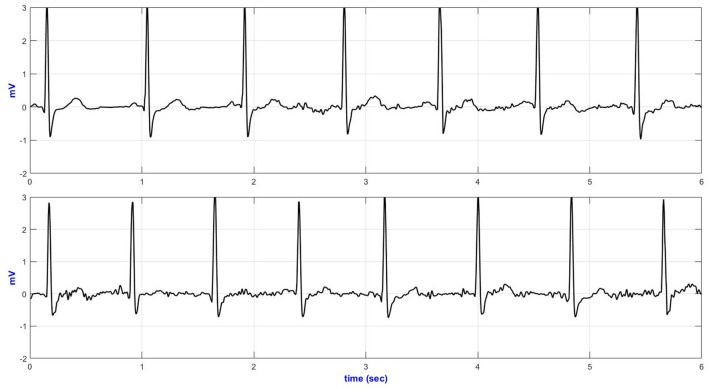
Lead-II representative ECG during up movement (top—wet textile electrodes and bottom—commercial wet-gel electrodes).

Quantitative signal quality parameters, including ApEn, basSQI, and SNR, were computed, and the results are presented in Table 4. The ECGs from the commercial wet-gel electrodes exhibited higher randomness (ApEn) and lower basSQI values compared to the wet-textile electrodes. However, ECGs from the commercial wet-gel electrodes showed higher SNR during deep breathing, sitting/standing from a chair, and climbing stairs (Table 4). Figure 5 presents a representative ECG acquired during up movement.

### Comparison Between the E-Textile ECG Monitor and the Commercial Holter Monitor

**Figure 6:**
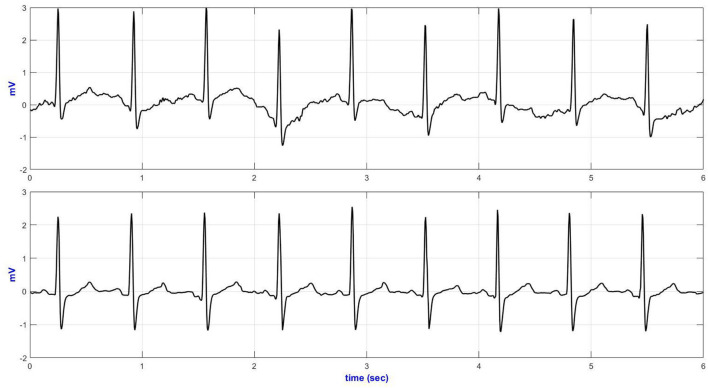
V5 representative ECG during climbing stairs (top—Holter ECG and bottom—textile ECG monitor).

Six seconds of representative ECG trances from the Holter and textile-based ECG are presented in Figure 6. Increased body movement (e.g., climbing stairs, Figure 6) forced the ECG to drift away from the isoelectric line.

Table 5 compares the quantitative signal quality parameters. The Holter ECG revealed an increased interference in the low-frequency region of the ECG acquired, especially during sitting/standing activities (basSQI = 0.8067), lying on a bed (basSQI = 0.8687), climbing stairs (basSQI = 0.8874), and making a phone call from a mobile phone (basSQI = 0.9325). The significantly higher ApEn values of the ECGs from the reference ambulatory monitor (Table 5) supported the increased randomness of the Holter ECGs compared to the ECGs from the proposed textile-based ECG monitor.

**Table 5:** SQI analysis results of lead V5 ECG from the reference Holter monitor (using wet-gel commercial electrodes) and the textile ECG monitor (using textile electrodes).

**Body movement / activities**	**ApEn descriptive statistics (Mean** **±** **SD)**		**basSQI**		**SNR**	
	**Holter monitor**	**Textile ECG monitor**	**Holter monitor**	**Textile ECG monitor**	**Holter monitor**	**Textile ECG monitor**
Deep breathing	**0.1385** **±** 0.0245^*^	0.1268 ± 0.0126	0.9818	0.9898	20.1131	18.7119
Coughing	**0.1590** **±** 0.0197^*^	0.1328 ± 0.0093	0.9639	0.9890	15.3232	16.8440
Sideways	**0.1665** **±** **0.0520**	0.1459 ± 0.0329	0.9896	0.9904	13.6312	18.4488
Up	**0.1138** **±** **0.0114**	0.1097 ± 0.0063	0.9862	0.9911	15.5721	16.0624
Sitting	**0.1294** **±** 0.0274^*^	0.1143 ± 0.0102	0.9896	0.9867	16.0861	18.1898
A phone call	**0.1842** **±** 0.0191^*^	0.1244 ± 0.0156	***0.9325***	0.9920	14.6479	15.3835
Sitting / standing	**0.1803** **±** 0.0353^*^	0.1400 ± 0.0184	***0.8067***	***0.9287***	9.1645	16.2594
Lying on a bed	**0.1442** **±** 0.0222^*^	0.1126 ± 0.0052	***0.8687***	0.9924	13.7291	15.0579
Stairs	**0.17871** **±** 0.0224^*^	0.1268 ± 0.0126	***0.8874***	0.9970	11.9310	17.0698

*Sideways, moving the hands sideways and moving them back to the midline horizontally; up, raise arms above the head and moving them back; sitting / standing, sitting / standing from a chair; stairs, climbing stairs; ApEn, Approximate entropy; basSQI, baseline power signal quality index; SNR, Signal to Noise Ratio; Bold italic - Red, moderate to intense lower frequency noise (basSQI <0.95); Bold, higher ApEn value*.

* *Statistically significant (p < 0.05) compared to the ECG from the Textile ECG monitor*.

The SNR analysis (Table 5) further confirmed the higher baseline drifts within the reference Holter monitor during the two activities (sitting/standing from a chair and climbing stairs) compared to the ECG acquired from the proposed textile-based ECG monitor during the same sequence of body movements. However, the textile ECGs collected during sideways movement showed lower signal power.

Table 6 summarizes the QRS duration and QTc measurements from ECGs collected from both the reference Holter monitor and the proposed textile-based ECG monitor. There was no difference in the number of normal QRS intervals between the two systems. However, the QT_C_ measures were different between the two systems when the subject was sitting in a chair. For the signals from the Holter monitor, 58 of the 262 QT_C_ intervals were identified as long QT_C_ (>0.45 s), and one was a short QT_C_ interval (<0.36 s). However, 261 of the 262 QT_C_ intervals from the textile-based ECG were detected as normal (Table 6 top). For both the Holter monitor and the textile-based ECG, one QT_C_ interval was missing. The ECG was then denoised using a 2nd order high pass Butterworth filter (fc = 0.67 Hz) in a MATLAB environment and the QT_C_ intervals were computed again. All 58 QT_C_ intervals identified as long QT_C_ within the reference ambulatory monitor were classified as normal QT_C_ after denoising. Moreover, the missed T-wave was recovered in both the Holter and textile ECGs (Table 6 bottom).

**Table 6:** Summary of the QRS duration and QT_C_ intervals of ECG from the Holter monitor and textile-based ECG monitor during different body movements and activities of the daily living.

**Body movement / activities**	**Reference Holter ECG (mV5)**					**Textile based ECG (V5)**				
	**QRS duration**		**QT_C_**			**QRS duration**		**QT_C_**		
	**NQRS**	**LQRS**	**NQT_C_**	**SQT_C_**	**LQT_C_**	**NQRS**	**LQRS**	**NQT_C_**	**SQT_C_**	**LQT_C_**
Deep breath	333	0	332	1	0	333	0	333	0	0
Coughing	159	0	159	0	0	159	0	159	0	0
Sideways	147	0	146	1	0	147	0	146	0	1
Up	140	0	140	0	0	140	0	140	0	0
Sitting	261	1	202	1	58	262	0	261	0	0
A phone call	82	0	79	3	0	82	0	82	0	0
Sitting / standing	78	0	77	1	0	78	0	77	0	1
Lying on a bed	70	0	70	0	0	70	0	69	1	0
Stairs	160	0	158	0	2	160	0	160	0	0
**Body movement / activities**	**QT**_C_ **after the ECGs were denoised**									
	**Reference Holter ECG (mV5)**					**Textile based ECG (V5)**				
	**QRS duration**		**QT**_C_			**QRS duration**		**QT**_C_		
	**NQRS**	**LQRS**	**NQT**_C_	**SQT**_C_	**LQT**_C_	**NQRS**	**LQRS**	**NQT**_C_	**SQT**_C_	**LQT**_C_
Sitting	262	0	261	1	0	262	0	262	0	0

*Sideways, moving the hands sideways and moving them back to the midline horizontally; up, raise arms above the head and moving them back; sitting / standing, sitting / standing from a chair; stairs, climbing stairs; QT_C_, corrected QT interval; NQRS, number of the normal (0.08–0.12 s); LQRS, long QRS (>0.12 s) durations; NQT_C_, number of the normal (0.36–0.45 s); SQT_C_, short (<0.36 s); LQT_C_, long QT_C_ (>0.45 s) intervals, respectively. The textile-based ECG showed higher accuracy compared to the reference Holter monitor*.

## Discussion

The aim of this study was to determine whether signals produced by an e-textile ECG monitor with textile electrodes in an EASI configuration are of sufficient quality to be used for cardiac monitoring. Specific objectives were to investigate the effect of the textile electrode characteristics, placement, and condition on signal quality, and finally to compare results to a reference ECG obtained from a current clinical standard the Holter monitor.

### Textile Electrode Characteristic

The relation between the size of the electrodes and the ECG quality was studied. Results showed that the bigger the textile electrodes' size, the better the signal quality and the lower the approximate entropy (randomness of the signal). This finding is in agreement with that reported by Ueno et al. (33). Throughout the experiment, the ECG from the 70 mm^2^ resulted in a higher peak ECG signal for all body movements and daily activities except for deep breathing, where the ECG collected *via* the 60 mm^2^ textile electrodes showed slightly higher SNR. The increased ECG amplitude and higher signal power for an increased electrode area also agree with previous studies (34–36).

In a previous study, Cömert and Hyttinen (37) used a 4 mm thick cushion padding structure to support their textile electrodes. The authors showed that the electrode support structure and padding increased the stability of the skin-electrode interface and distributed the compressive force uniformly across the electrode. Moreover, a soft support structure has been shown to produce less noise as it allows the textile electrode to follow the underlying anatomy (37). In this study, 3, 5, and 10 mm thick textile electrodes were constructed using a soft support structure made of Statfree® conductive polyurethane foam.

Throughout the trial, ECG signals from the 3 and 5 mm textile electrodes showed higher signal power, lower randomness, and decreased motion artifact aligning with Comert and Hyttinen's 2015 study (37), where they showed a positive relationship between electrode padding and signal quality using a 4 mm thick padding. In another study, Cömert et al. (38) examined the effect of different thicknesses and types of padding using 6, 9, 13, 14, and 16 mm thick electrodes where the padding was made of two different grades of SunMate memory foam and Poron XRD impact protection cushion. The authors reported the positive effect of padding on signal quality. However, the padding that resulted in the best ECG quality was not clearly stated.

In this study, increasing the padding thickness beyond 5 mm showed decreased signal quality. For example, during climbing stairs, the ECG from the 10 mm thick textile sensors performed worst. This may be a result of the thicker textile electrodes (thicker padding, e.g., 10 mm thick textile electrodes) shifting position and sliding when subjected to movement more so than the thinner (e.g., 3 and 5 mm) textile electrodes. Moreover, Cömert et al. (38) used a different technique to acquire the ECG (electrode was placed on the upper arm and was subjected to different magnitude pressure from 5 to 25 mmHg) and a different material to make the support structure. In our experiment, the electrodes were placed along the EASI configuration, and the support structure was made of Statfree® conductive polyurethane foam. Hence, it is difficult to compare the results directly.

### Electrode Placement

Based on the EASI configuration, placing the “A” and “I” electrodes at the anterior axillary line showed a lower ApEn. At the same time, during sideways and up movement, the medial axillary and posterior “AI” placement showed higher randomness (an increased ApEn) in the acquired ECG. As the hands were moved side to side (sideways) and raised above the head and then moved back (up movement), there was a high chance of the arms touching the electrodes placed under the armpit and on the posterior axillary lines, resulting in an unstable skin-electrode interface. This continuous impedance-change induced low-frequency interference in the acquired ECGs.

Moreover, moving the electrodes from the anterior-axillary to the posterior-axillary line diminished the R-wave ECG amplitude, reduced the power contained within the acquired ECG, and increased low-frequency noise. From an electrophysiology perspective, where the body is assumed to be a volume conductor (39), the further the sensors from the source (the heart), the higher the impedance of the volume conductor (39, 40). Therefore, it is unsurprising that the amplitude of the ECG collected with the AI electrodes on the medial axillary line is greater than the ECG collected at the posterior axillary line.

### Electrode Condition

Wet textile electrodes (from sweating) were compared to dry counterparts. They were found to perform better as the dry textile electrodes drift easily, change position, and are susceptible to motion artifact during physical activities. Moreover, the performance of the wet textile electrodes was comparable to that of commercial wet-gel electrodes. Previous studies support this result. Pani et al. (41) used poly (3,4-ethylene dioxythiophene): poly (styrene sulfonate) textile electrodes to compare dry textile electrodes, wet textile electrodes, and commercial Ag/AgCl electrodes during different daily activities. The authors showed that the dry textile electrodes performed poorly, especially during physical activities. However, the wet textile electrodes were as good as the Ag/AgCl commercial electrodes. When evaluated based on a QRS detector, the wet textile electrodes performed better than the commercial Ag/AgCl electrodes.

Marozas et al. (34) compared commercial Ag/AgCl electrodes to wet textile electrodes in exercise ECG. The authors concluded that the textile electrodes showed significant noise in the low-frequency band (0–0.67 Hz) while textile electrodes are less prone to broadband noise (0–250 Hz) compared to the Ag/AgCl electrodes. We did not see the same level of low-frequency noise; however, results cannot be directly compared as we did not experiment on exercise ECG. Also, Marozas et al. (34) used three electrodes placed on the thorax area 25 cm apart, where in our case we used the EASI electrode configuration. However, the analog front-end of our hardware has been carefully designed to minimize low-frequency distortion, which might be why we did not observe intense low-frequency noise from the wet textile electrodes.

### Comparison Between the E-Textile ECG Monitor and the Commercial Holter Monitor

The performance of the textile-based ECG monitor was compared against the traditional Holter monitor. Channel one (modified V5) from the Holter monitor and the V5 ECG from the textile-based ECG monitor were used to analyze the data. In the time domain plots, there was no significant difference between the ECGs acquired from the Holter monitor and the textile-based ECG monitor. Even from the noisy recording, it was possible to identify the QRS complexes. The main problem seen on the time traces were baseline drift. In both the Holter monitor and the textile-based ECG monitor the motion artifact within the QRS band (5–15 Hz) was minimal as confirmed by the SQI values. However, the ECGs from the Holter monitor showed an increased low-frequency noise, and hence lower basSQI values during a phone call, sitting / standing from a chair, lying on a bed, and climbing stairs.

In summary, compared to the body movements (e.g., deep breathing), the daily activities (e.g., sitting / standing from a chair) resulted in greater low-frequency interference within the ECG acquired from the Holter monitor. The reference ambulatory monitor and the smart ECG vest were used simultaneously. In this regard, for an increased activity like sitting / standing from a chair, the smart ECG vest might be touching the Holter lead wires and hence introducing an increased noise within the Holter ECG.

The precise delineation of the QRS duration and QT_C_ interval is important to detect cardiac episodes. In this regard, the QRS durations and the QT_C_ intervals were extracted from the Holter ECGs and the textile-based ECGs, and the results compared. Based on the QRS and QT_C_ analysis, there was no significant difference between the Holter monitor and the textile-based ECG monitor. However, the textile-based ECG monitor showed higher accuracy than the Holter monitor for the ECG collected when the participant sat quietly. Previous studies showed that ECGs acquired during upright position showed a decreased amplitude in the ST-segments (42), T (43), and Q (44) waves. Moreover, Yokus and Jur (45) compared the textile and wet-commercial electrodes and reported that ECGs collected from textile electrodes during sitting showed a higher SNR. As a result, sitting ECGs from the Holter monitor might be prone to low-frequency noise that affects the lower amplitude Q and T waves. The peak detection algorithm might be an additional contributing factor (34, 46).

## Limitations

The major limitation of our study is that data were only collected from a single healthy participant. Even though ethics approval was obtained from the Flinders University Social and Behavioral Research Ethics Committee (SBREC: project code – 8490), it was not possible to recruit and collect data from more participants or cardiac patients due to the outbreak of the COVID-19 pandemic. As a result, given a unisex design of the ECG vest, the effect of different body sizes, body hair, skin type, and gender on ECG quality and the presence or absence of skin irritation due to textile electrodes were not studied.

## Conclusion and Implications for Further Research

This study reports on the testing and evaluation of a wireless and wearable EASI-based e-textile ECG monitor. Optimal electrodeposition remains an active area of research for quality ECG transduction. In this regard, the best electrodeposition for the EASI configuration was studied, where the results could be extended for the traditional EASI lead system ECG. Placing the “A” and “I” electrodes on the left and right anterior axillary point, respectively, showed higher signal quality compared to the standard EASI electrode placement.

The preliminary results revealed that there was no significant signal quality difference between the traditional Holter monitor and the e-textile ECG monitor. The standard ambulatory monitor utilizes sticky wet-gel electrodes where the ECG quality deteriorates over time due to the drying of the gel interface. Moreover, the ECG lead wires reduced the comfort of the users. On the other hand, the textile-based ECG monitor has embedded wires and textile electrodes.

The use of the EASI configuration combined with the wearable and wireless design of the e-textile ECG monitor could support long-term ambulatory monitoring of cardiac patients and increase access to cardiac rehabilitation *via* telemonitoring. The intuitive design of the ECG vest will significantly reduce the time needed to train the users. No assistance is required to put on/off the smart ECG vest. Therefore, it will also lower diagnosis errors due to misplaced electrodes. Further research is needed to validate the e-textile ECG monitor in a larger trial and on a cardiac population.

## Data Availability Statement

The raw data supporting the conclusions of this article will be made available by the authors, without undue reservation.

## Ethics Statement

The studies involving human participants were reviewed and approved by Flinders University Social and Behavioral Research Ethics Committee (SBREC: Project Code – 8490). The patients/participants provided their written informed consent to participate in this study.

## Author Contributions

MT conceived the original idea, contributed to the study design, analyzed the data, and wrote the manuscript with support from DH, RC, and KR. All authors contributed to the data analysis, interpretation, and to drafting the article.

## Funding

Flinders University supported this project. CINOP Global and Addis Ababa Institute of Technology supported MT through Nuffic funded NICHE project NICHE/ETH/246. Development of the ECG vest was supported by the Tom Simpson Trust Fund and the National Heart Foundation of Australia. KR was funding the publication cost.

## Conflict of Interest

The authors declare that the research was conducted in the absence of any commercial or financial relationships that could be construed as a potential conflict of interest.

## Publisher's Note

All claims expressed in this article are solely those of the authors and do not necessarily represent those of their affiliated organizations, or those of the publisher, the editors and the reviewers. Any product that may be evaluated in this article, or claim that may be made by its manufacturer, is not guaranteed or endorsed by the publisher.

## Footnotes

^1^Available at: https://archive.physionet.org/physiotools/ApEn/.

^2^Available at: http://apps.gehealthcare.com/servlet/ClientServlet.

## References

[1] Roth GA Johnson C Abajobir A Abd-Allah F Abera SF Abyu G. Global, regional, and national burden of cardiovascular diseases for 10 causes, 1990 to 2015. J Am Coll Cardiol. (2017) 70:1–25. 10.1016/j.jacc.2017.04.052 28527533 PMC5491406

[2] Loue S Sajatovic M. Encyclopedia of Immigrant Health. New York, NY: Springer Science and Business Media, LLC (2012). 10.1007/978-1-4419-5659-0

[3] Roth GA Abate D Abate KH Abay SM Abbafati C Abbasi N. Global, regional, and national age-sex-specific mortality for 282 causes of death in 195 countries and territories, 1980–2017: a systematic analysis for the Global Burden of Disease Study 2017. Lancet. (2018) 392:1736–88. 10.1016/S0140-6736(18)32203-7 30496103 PMC6227606

[4] Mukhopadhyay SC. Wearable Electronics Sensors: For Safe and Healthy Living. Berlin: Springer (2015). 10.1007/978-3-319-18191-2

[5] Single Lead Electrocardiography (ECG) Equipment - Global Market Outlook (2017-2026). Guinness Brewery, Dublin Ireland: Research and Markets. (2019). 4753127.

[6] Jolly K Lip GY Taylor RS Raftery J Mant J Lane D. The Birmingham Rehabilitation Uptake Maximisation study (BRUM): a randomised controlled trial comparing home-based with centre-based cardiac rehabilitation. Heart. (2009) 95:36–42. 10.1136/hrt.2007.127209 18332063

[7] Stoppa M Chiolerio A. Wearable electronics and smart textiles: a critical review. Sensors. (2014) 14:11957–92. 10.3390/s140711957 25004153 PMC4168435

[8] Teferra MN Ramos JS Fleury A Kourbelis C Newman P Hobbs D. E-textile electrocardiogram (ECG) monitoring in cardiac patients: a scoping review. JBI Database System Rev Implement Rep. (2019) 17:1958–98. 10.11124/JBISRIR-2017-003989 31633636

[9] Chamadiya B Mankodiya K Wagner M Hofmann UG. Textile-based, contactless ECG monitoring for non-ICU clinical settings. J Ambient Intell Humaniz Comput. (2013) 4:791–800. 10.1007/s12652-012-0153-8

[10] Fleury A Sugar M Chau T. E-textiles in clinical rehabilitation: a scoping review. Electronics. (2015) 4:173–203. 10.3390/electronics401017332279878

[11] Coosemans J Hermans B Puers R. Integrating wireless ECG monitoring in textiles. Sens Actuators A Phys. (2006) 130–131:48–53. 10.1016/j.sna.2005.10.052

[12] Tsukada YT Tokita M Murata H Hirasawa Y Yodogawa K Iwasaki Y-k. Validation of wearable textile electrodes for ECG monitoring. Heart Vessels. (2019) 34:1203–11. 10.1007/s00380-019-01347-8 30680493 PMC6556171

[13] Di Rienzo M Racca V Rizzo F Bordoni B Parati G Castiglioni P. Evaluation of a textile-based wearable system for the electrocardiogram monitoring in cardiac patients. Europace. (2013) 15:607–12. 10.1093/europace/eus368 23258818

[14] Feild DQ Feldman CL Hor BM. Improved EASI coefficients: their derivation, values, and performance. J Electrocardiol. (2002) 35:23–33. 10.1054/jelc.2002.37151 12539096

[15] Sundar A Pahwa V Das C Deshmukh M Robinson N. A comprehensive assessment of the performance of modern algorithms for enhancement of digital volume pulse signals. Int J Pharma Med Biol Sci. (2016) 5:91. 10.18178/ijpmbs.5.1.91-98

[16] Alfaouri M Daqrouq K. ECG signal denoising by wavelet transform thresholding. Am J Appl Sci. (2008) 5:276–81. 10.3844/ajassp.2008.276.281

[17] Raeiatibanadkooki M Quachani SR Khalilzade M Bahaadinbeigy K. Real time processing and transferring ECG signal by a mobile phone. Acta Informatica Medica. (2014) 22:389. 10.5455/aim.2014.22.389-392 25684847 PMC4315648

[18] Phukpattaranont P. Improvement of signal to noise ratio (SNR) in ECG signals based on dual-band continuous wavelet transform. In: Signal Inf Process Assoc Annu Summit Conf APSIPA Asia Pac. Siem Reap: IEEE (2014). p. 1–4. 10.1109/APSIPA.2014.7041610

[19] Pincus S. Approximate entropy (ApEn) as a complexity measure. Chaos Interdiscipl J Nonlin Sci. (1995) 5:110–7. 10.1063/1.166092 12780163

[20] Moody GBM. Approximate Entropy (ApEn): PHYSIONET. (2015). Available online at: https://archive.physionet.org/physiotools/ApEn/ (accessed August 26, 2019). 10.1007/978-1-4614-6675-8_496

[21] Pincus SM Goldberger AL. Physiological time-series analysis: what does regularity quantify? Am J Physiol Heart Circ Physiol. (1994) 266:H1643–56. 10.1152/ajpheart.1994.266.4.H1643 8184944

[22] Clifford G Behar J Li Q Rezek I. Signal quality indices and data fusion for determining clinical acceptability of electrocardiograms. Physiol Meas. (2012) 33:1419. 10.1088/0967-3334/33/9/1419 22902749

[23] Wang NC Maggioni AP Konstam MA Zannad F Krasa HB Burnett JC. Clinical implications of QRS duration in patients hospitalized with worsening heart failure and reduced left ventricular ejection fraction. J Am Med Assoc. (2008) 299:2656–66. 10.1001/jama.299.22.2656 18544725

[24] Gold MR Thébault C Linde C Abraham WT Gerritse B Ghio S. Effect of QRS duration and morphology on cardiac resynchronization therapy outcomes in mild heart failure: results from the Resynchronization Reverses Remodeling in Systolic Left Ventricular Dysfunction (REVERSE) study. Circulation. (2012) 126:822–9. 10.1161/CIRCULATIONAHA.112.097709 22781424

[25] Ambhore A Teo S-G Omar ARB Poh K-K. ECG series. Importance of QT interval in clinical practice. Singapore Med J. (2014) 55:607. 10.11622/smedj.2014172 25630313 PMC4291996

[26] Trinkley KE Lee Page R Lien H Yamanouye K Tisdale JE. QT interval prolongation and the risk of torsades de pointes: essentials for clinicians. Curr Med Res Opin. (2013) 29:1719–26. 10.1185/03007995.2013.840568 24020938

[27] Zipes DP Libby P Bonow RO Mann DL Tomaselli GF. Braunwald's Heart Disease: A Textbook of Cardiovascular Medicine. 11 ed. Philadelphia, PA: Elsevier Health Sciences (2019).

[28] Sedghamiz H. BioSigKit: a Matlab toolbox and interface for analysis of biosignals. J Open Source Softw. (2018) 3:671. 10.21105/joss.00671

[29] Albert DE. Methods and systems for electrode placement. Google Patents. (2015).

[30] General Electric Company. SEER™ 1000 ECG Recorder Mobile Application Operating Manual. General Electric Company (2016). Available online at: http://apps.gehealthcare.com/servlet/ClientServlet (accessed March 13, 2017).

[31] Lee S Kruse J. Biopotential electrode sensors in ECG/EEG/EMG systems. Analog Devices. (2008) 200:1–2. Available online at: https://www.analog.com/en/technical-articles/biopotential-electrode-sensors-ecg-eeg-emg.html (accessed February 18, 2019).

[32] Altman DG. Practical Statistics for Medical Research. Boca Raton, FL: CRC Press (1990). 10.1201/9780429258589

[33] Ueno A Akabane Y Kato T Hoshino H Kataoka S Ishiyama Y. Capacitive sensing of electrocardiographic potential through cloth from the dorsal surface of the body in a supine position: a preliminary study. IEEE Trans Biomed Eng. (2007) 54:759–66. 10.1109/TBME.2006.889201 17405385

[34] Marozas V Petrenas A Daukantas S Lukosevicius A. A comparison of conductive textile-based and silver/silver chloride gel electrodes in exercise electrocardiogram recordings. J Electrocardiol. (2011) 44:189–94. 10.1016/j.jelectrocard.2010.12.004 21353065

[35] Puurtinen MM Komulainen SM Kauppinen PK Malmivuo JA Hyttinen JA. Measurement of noise and impedance of dry and wet textile electrodes, and textile electrodes with hydrogel. In: IEEE Biomed Circuits Syst Conf. New York, NY: IEEE (2006). p. 6012–5. 10.1109/IEMBS.2006.260155 17946734

[36] Wu W Pirbhulal S Sangaiah AK Mukhopadhyay SC Li G. Optimization of signal quality over comfortability of textile electrodes for ECG monitoring in fog computing based medical applications. Future Gener Comput Syst. (2018) 86:515–26. 10.1016/j.future.2018.04.024

[37] Cömert A Hyttinen J. Investigating the possible effect of electrode support structure on motion artifact in wearable bioelectric signal monitoring. Biomed Eng Online. (2015) 14:1–18. 10.1186/s12938-015-0044-2 25976349 PMC4432977

[38] Cömert A Honkala M Hyttinen J. Effect of pressure and padding on motion artifact of textile electrodes. Biomed Eng Online. (2013) 12:26. 10.1186/1475-925X-12-26 23565970 PMC3637835

[39] Malmivuo J Plonsey R. Bioelectromagnetism: Principles and Applications of Bioelectric and Biomagnetic Fields. Oxford: Oxford University Press (1995). 10.1093/acprof:oso/9780195058239.001.0001

[40] Gacek A Pedrycz W. ECG Signal Processing, Classification and Interpretation: A Comprehensive Framework of Computational Intelligence. Berlin: Springer Science & Business Media (2011). 10.1007/978-0-85729-868-3_3

[41] Pani D Dessì A Saenz-Cogollo JF Barabino G Fraboni B Bonfiglio A. Fully textile, PEDOT: PSS based electrodes for wearable ECG monitoring systems. IEEE Trans Biomed Eng. (2015) 63:540–9. 10.1109/TBME.2015.2465936 26259215

[42] Sutherland DJ McPherson DD Spencer CA Armstrong CS Horacek BM Montague TJ. Effects of posture and respiration on body surface electrocardiogram. Am J Cardiol. (1983) 52:595–600. 10.1016/0002-9149(83)90033-4 6613884

[43] Khare S Chawala A. Effect of change in body position on resting electrocardiogram in young healthy adults. Nig J Cardiol. (2016) 13:125. 10.4103/0189-7969.187711

[44] Riekkinen H Rautaharju P. Body position, electrode level, and respiration effects on the Frank lead electrocardiogram. Circulation. (1976) 53:40–5. 10.1161/01.CIR.53.1.40 127674

[45] Yokus MA Jur JS. Fabric-based wearable dry electrodes for body surface biopotential recording. IEEE Trans Biomed Eng. (2015) 63:423–30. 10.1109/TBME.2015.2462312 26241969

[46] Weippert M Kumar M Kreuzfeld S Arndt D Rieger A Stoll R. Comparison of three mobile devices for measuring R–R intervals and heart rate variability: Polar S810i, Suunto t6 and an ambulatory ECG system. Eur J Appl Physiol. (2010) 109:779–86. 10.1007/s00421-010-1415-9 20225081

